# Simple and Fast One-Pot Cellulose Gel Preparation in Aqueous Pyrrolidinium Hydroxide Solution–Cellulose Solvent and Antibacterial Agent

**DOI:** 10.3390/polym13121942

**Published:** 2021-06-11

**Authors:** Elisabeth R. D. Seiler, Kohei Koyama, Tomoyuki Iijima, Tamao Saito, Yuko Takeoka, Masahiro Rikukawa, Masahiro Yoshizawa-Fujita

**Affiliations:** Department of Materials and Life Sciences, Sophia University, 7-1 Kioi-cho, Chiyoda-ku, Tokyo 102-8554, Japan; e-seiler@eagle.sophia.ac.jp (E.R.D.S.); kouheihunpty@icloud.com (K.K.); jima-tomo@eagle.sophia.ac.jp (T.I.); tasaito@sophia.ac.jp (T.S.); y-tabuch@sophia.ac.jp (Y.T.); m-rikuka@sophia.ac.jp (M.R.)

**Keywords:** cellulose, pyrrolidinium hydroxide, epichlorohydrin, cellulose gels, hydrogels, antibacterial activity

## Abstract

Cellulose is the main component of biomass and is the most abundant biopolymer on earth; it is a non-toxic, low-cost material that is biocompatible and biodegradable. Cellulose gels are receiving increasing attention as medical products, e.g., as wound dressings. However, the preparation of cellulose hydrogels employing unmodified cellulose is scarcely reported because of the cumbersome dissolution of cellulose. In previous studies, we developed the new promising cellulose solvent *N*-butyl-*N*-methylpyrrolidinium hydroxide in an aqueous solution, which can dissolve up to 20 wt% cellulose within a short time at room temperature. In this study, we employed this solvent system and investigated the gelation behavior of cellulose after crosslinker addition. The swelling behavior in water (swelling ratio, water uptake), the mechanical properties under compression, and the antibacterial activity against *Escherichia coli* and *Bacillus subtilis* were investigated. We have developed a simple and fast one-pot method for the preparation of cellulose gels, in which aqueous pyrrolidinium hydroxide solution was acting as the solvent and as an antibacterial reagent. The pyrrolidinium hydroxide content of the gels was controlled by adjustment of the water volume employed for swelling. Simple recovery of the solvent system was also possible, which makes this preparation method environmentally benign.

## 1. Introduction

For a sustainable society, the consumption of non-renewable resources, such as petroleum, must be reduced, making lignocellulosic biomass increasingly important as it is renewable and the most abundantly available raw material on the earth. The main component of lignocellulosic biomass and the most abundant biopolymer on earth is cellulose [1]. Materials based on cellulose are attractive because cellulose is a low-cost, non-toxic [2], biocompatible [3], and biodegradable [4] polymer that can be considered as an inexhaustible feedstock due to its renewability [1]. Cellulose hydrogels aroused extensive interest in a variety of fields because of their inherent biocompatibility [5], biodegradability [6], and their tissue-mimicking [7] properties. Hydrogels are based on a three-dimensional polymeric structure and can absorb and release large amounts of water or biological fluids [8]. Cellulose has plentiful hydrophilic functional groups (hydroxyl groups) in its backbone, which makes it promising for highly absorbent hydrogel systems. Due to their similar properties to tissue, there are approaches for the application of hydrogels from natural polymers (e.g., cellulose) as biomedical material, such as wound-dressing [9,10,11], scaffolds in tissue engineering [12,13] or drug-delivery systems [14]. The high water absorbency of hydrogels leads to many more possible applications in different areas, such as water reservoirs in agriculture [15], superabsorbers in personal health care products such as diapers or hygiene products [16], and among many others.

However, most cellulose hydrogels are based on cellulose derivatives, for instance, methyl cellulose (MC) [17], hydroxypropylmethyl cellulose (HPMC) [12], carboxymethyl cellulose (CMC) [10,11], hydroxypropyl cellulose (HPC) [18], or hydroxyethyl cellulose (HEC) [19]. The dissolution of these cellulose derivatives is improved in comparison to non-modified cellulose. However, chemical modification leads not only to additional steps, time, and costs for preparation, but the environmental impact of the chemicals and solvents required for the derivatization must also be taking into consideration. Therefore, the ideal preparation method for cellulose hydrogels should be native, unmodified cellulose.

The preparation of hydrogels can basically be divided into two categories: physical and chemical cross-linking. Compared to physically cross-linked hydrogels, chemically cross-linked hydrogels generally exhibit adjustable degradation behavior, increased stability, and mechanical properties [20]. Therefore, this study focuses on chemically cross-linked cellulose hydrogels.

Preparation methods of cellulose hydrogels employing native cellulose are scarcely reported because of the usually cumbersome dissolution of cellulose due to strongly pronounced inter-and intramolecular hydrogen bonds. Common solvents for cellulose, e.g., *N*-methylmorpholine *N*-oxide (NMMO) aqueous solution [21], NaOH/thiourea aqueous solution [22], *N*,*N*-dimethylacetamide/LiCl [23], are often toxic and require heating or cooling/freezing conditions which are time and energy-consuming. By employing other compounds as solvents, some dissolution parameters can be improved according to the literature. Zhou et al. dissolved unmodified cellulose in an aqueous solution of NaOH/urea as solvent [24]. No heating was applied for the dissolution. However, cooling conditions (−5 to −10 °C for 12 h) were required. Moreover, heating to 50 °C for 4 to 20 h was required for cross-linking with the crosslinker epichlorohydrin (ECH) and curing of the gel [24]. Another study showed that cross-linking with ECH was also possible at low temperatures (5 °C 24 h) [25]. Other studies about native cellulose hydrogels are prepared by using the solvent mixture lithium chloride and *N*-methyl-2-pyrrolidinone (LiCl/NMP) with carboxylic anhydrides as a crosslinker, e.g., 1,2,3,4-butanetetracarboxylic dianhydride (BTCA) [26] or succinic anhydride (SA) [27]. Even though cellulose can be dissolved in LiCl/NMP at 25 °C, stirring for 48 h was required. In addition, nucleophilic catalysis with 4-dimethylaminopyridine (DMAP) is required for cross-linking with carboxylic anhydrides (stirring for 24 h at 25 °C).

In summary, the current methods for the preparation of cellulose hydrogels based on unmodified cellulose are still cumbersome, firstly, because of the dissolution of cellulose (temperature, time, partly toxic solvents), and secondly, because of the cross-linking and gel curing (temperature, time, additional reagents for catalysis). Thus, the goal of this study was to investigate a simple, fast, and environmentally benign preparation method for unmodified cellulose gels under ambient conditions.

Applications of hydrogels from natural resources are conceivable in the field of biomaterials. However, direct or close contact with the skin, e.g., as a diaper or wound dressing, can lead to infections. Bacterial infections can be effectively inhibited if the employed gels have antibacterial activity. Cellulose composites that have been used as antibacterial wound dressings include MC-alginate-gallium composites [28], CMC-keratin hydrogels with clindamycin [29], CMC fibers linked with ε-poly-L-lysine [30], carboxylated cellulose nanofibers with gelatin and aminated silver nanoparticles [31], HEC with tungsten oxide [32], bacterial cellulose with *Bacillus subtilis* (*B. subtilis*) [33], or with silver nanoparticles [31,34]. Most of these composites, however, are based on cellulose derivatives, whereby the environmental friendliness of the derivatization process must be questioned, or the composites consist of bacterial cellulose, which does not belong to the abundant lignocellulosic biomass because it is produced by bacteria. Antibacterial agents for cellulose hydrogels are, e.g., gallium [28], silver nanoparticles [34,35], zinc oxide [36], tungsten, oxide [32], chloramphenicol [5], clindamycin [29], ε-poly-l-lysine [30], or other bacteria [33]. However, loading of the antibacterial agent requires extra steps, and the binding affinity with the surface of hydrogels and the release of the agent can be problematic [37]. If the binding affinity is low or the release is rapid, skin irritation may occur, and the effectiveness of the antibacterial agent will be short-lived. A simple and fast preparation method for antibacterial gels, which does not require extra loading, would be favorable.

**Scheme 1:**
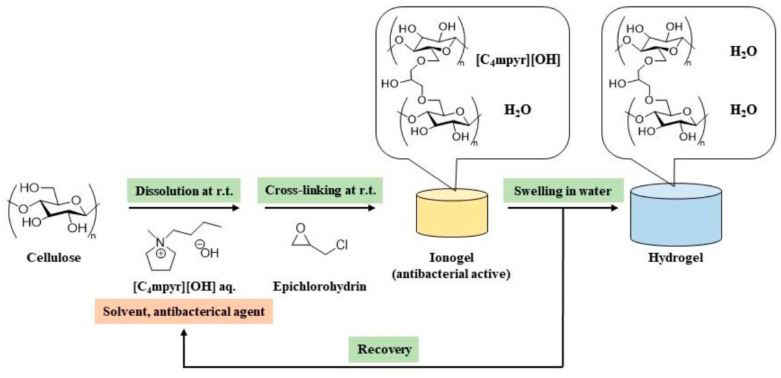
Concept of this research. After dissolution and cross-linking at room temperature (r.t.), the obtained gels (ionogels) were imbibed in water to obtain hydrogels, trying to recover the solvent. The mechanical, antibacterial, and swelling behavior of the ionogels and hydrogels were investigated.

Due to the numerous possible applications for cellulose hydrogels, it is necessary to develop environmentally friendly, simple, efficient, and cost-effective technologies for the preparation of cellulose gels. In this study, we concentrated on a simple, fast, and environmentally benign procedure for preparing cellulose gels by employing unsubstituted cellulose. In previous studies, we found that *N*-butyl-*N*-methylpyrrolidinium hydroxide in an aqueous solution ([C_4_mpyr][OH] aq.) enables the dissolution of up to 20 wt% cellulose within a short time at room temperature [38]. Since no heating or cooling conditions are required, energy consumption for dissolution is low, making it a more environmentally benign solvent for cellulose processing. The co-solvent water is also an abundant, non-toxic compound which makes the dissolution process even more cost-efficient and environmentally friendly. ECH was chosen as the crosslinker because it is a highly reactive etherifying agent that can cross-link the hydroxide groups of cellulose under alkaline conditions without catalysis, even at low temperatures [24,25]. After developing a simple one-pot preparation method for unmodified cellulose gels by employing [C_4_mpyr][OH] in aqueous solution and ECH, the swelling ratio, water uptake, mechanical properties under compression as well as the antibacterial activity of the cellulose gels were investigated. In this study, the antibacterial property of gels with and without [C_4_mpyr][OH] was investigated by using the disk-diffusion test to evaluate possible applications of the gels as a biomedical material. The disk diffusion method is a common evaluation method for antimicrobial activity, where the microbial growth inhibition is expressed as a diameter [39,40]. Furthermore, attempts were made to recover the solvent [C_4_mpyr][OH] in aqueous solution in order to enable an environmentally friendly recycling process. The concept of this research is summarized in Scheme 1.

## 2. Materials and Methods

### 2.1. Materials

Microcrystalline Avicel^®^ PH-101 cellulose was purchased from Sigma-Aldrich (St. Louis, MO, USA) and was dried at 80 °C under vacuum for 12 h before use. *N*-Butyl-*N*-methylpyrrolidinium chloride (>99%) was also purchased from Sigma-Aldrich (St. Louis, MO, USA). Silver oxide (99%) and activated charcoal powder were purchased from FUJIFILM Wako Pure Chemical Corp. (Chuo-ku, Osaka, Japan). Epichlorohydrin (ECH) (>99%) and tetramethylsilane (99%) were purchased from Tokyo Chemical Industry Co., Ltd. (Chuo-ku, Tokyo, Japan). Dimethyl sulfoxide-*d*_6_ (DMSO-*d*_6_) (99.9% D) was purchased from Kanto Chemical Co., Inc. (Chuo-ku, Tokyo, Japan). Unless otherwise specified, reagents and chemicals were used as received without further purification.

### 2.2. Synthesis of [C_4_mpyr][OH] Aqueous Solution

Following the procedure of Sun et al., *N*-butyl-*N*-methylpyrrolidinium chloride (107 mmol, 1 equivalent (eq.)) and silver oxide (64 mmol, 0.6 eq.) were stirred in 200 mL deionized water at room temperature, whereby the stirring time was changed from 6 h to 1 h [41]. The obtained yellow liquid was purified with activated charcoal powder to remove the color. After the evaporation of water, a colorless viscous liquid was obtained (yield: 90%). The water content was measured volumetrically by Karl-Fischer titration, and the content was adjusted whether by addition or evaporation of water until a solution with 50 wt% water was obtained. ^1^H NMR (400 MHz, DMSO-*d*_6_) *δ* = 3.39–3.52 (m, 4H), 3.32–3.28 (m, 2H), 2.98 (s, 3H), 2.07 (s, 4H), 1.63–1.71 (m, 2H), 1.31 (td, J = 14.8, 7.5 Hz, 2H), 0.90–0.94 (t, J = 7.4 Hz, 3H,). MS (EI, 70 eV): *m/z* (%) = 446.3, 305.1, 152.0, 142.1 ([P14]^+^).

### 2.3. Characterization of [C_4_mpyr][OH] Aqueous Solution

[C_4_mpyr][OH] aqueous solution was diluted in DMSO-*d*_6_ with tetramethylsilane as reference. It was characterized by ^1^H NMR spectroscopy on a Bruker AVANCE III HD NanoBay 400 MHz NMR spectrometer from Bruker Corporation (Billerica, MA, USA) at 25 °C. Mass spectra were measured on a JEOL JMS-SX 102A mass spectrometer from JEOL Ltd. (Akishima-shi, Tokyo, Japan). The water content was determined in triplicates through Karl Fischer titration using a Kyoto Electronics Manufacturing MKH-710M titrator from Kyoto Electronics Manufacturing Co., Ltd. (Minami-ku, Kyoto, Japan), and the average was used. The titration performance was regularly checked with KEMAQUA Water Standard 10 from Kyoto Electronics Manufacturing Co., Ltd. (Minami-ku, Kyoto, Japan) containing 1% water to adjust the titration factor. Throughout the experiments, a Shimadzu AP 224X from Shimadzu Corporation (Nakagyo-ku, Kyoto, Japan) weighing scale (d = 0.1 mg) was used.

### 2.4. Dissolution of Microcrystalline Cellulose

Dried microcrystalline cellulose (0.1, 0.15, 0.2, and 0.3 g (5, 7.5, 10, and 15 wt%)) was added each into aqueous [C_4_mpyr][OH] solutions (2.0 g) with 50 wt% water. It was mixed with a vortex mixer for 10 s and stirred for 10 min at 250 rpm on an EYELA RCH-1000 heat stirrer plate at 25 °C from Tokyo Rikakikai Co., Ltd. (Bunkyo-ku, Tokyo, Japan). In the case of inhomogeneity, a spatula was used to reduce the cellulose coagulates. Stirring was followed by sonication for 10 min to reduce air bubbles.

### 2.5. Gelation Test

A certain amount of crosslinker (ECH) was added with a micropipette to completely dissolved cellulose solutions to determine the gelation behavior. The molar ratio of ECH to anhydroglucose unit (AGU) of cellulose for each sample was 1, 2, and 3 eq. A timer was started after inserting ECH, and it was mixed with a vortex mixer for 10 s, followed by stirring for 30 s at 250 rpm to ensure homogeneous distribution of the crosslinker. Complete gelation was defined as the time where no fluidity was observable. The timer was stopped when the samples reached the self-standing state. This experiment was conducted three times, and the average value was used.

As a negative control, 10 wt% ECH was added to 2.0 g [C_4_mpyr][OH] (50 wt% water) without cellulose to confirm that no gelation occurs between [C_4_mpyr][OH] and ECH. It was stirred at 250 rpm at 25 °C. If no visual gelation occurred after 1 h, 10 wt% ECH was added to the solution until a maximum amount of 30 wt% ECH.

### 2.6. Preparation of Cellulose Gels (Ionogels and Hydrogels)

After dissolving 5, 7.5, 10, and 15 wt% dried microcrystalline cellulose in 2.0 g [C_4_mpyr][OH] aqueous solution directly in a mold, 3 eq. of ECH was added directly to each transparent solution. It was mixed with a vortex mixer for 10 s, followed by stirring for 30 s at 250 rpm at 25 °C. The samples were left on the heat plate until they were self-standing, depending on the cellulose content, for 3–10 min. The obtained gels were called ionogels and had a cylindrical shape (19 mm diameter × 9 mm height). Hydrogels were obtained by placing the ionogels in 20 mL water for 24 h at room temperature to extract [C_4_mpyr][OH] from the gel. The water was changed 2, 4, 5, or 9 times with a total water volume of 40, 80, 100, or 180 mL, depending on the experiment.

### 2.7. [C_4_mpyr][OH]-Content of the Cellulose Gels

The cellulose gels that were imbibed in 40, 80, or 180 mL water were freeze-dried at −50 °C under vacuum for 12 h. Elemental analysis of the obtained cryogels was performed to determine the [C_4_mpyr][OH]-content via nitrogen content. This evaluation was performed in triplicates, and the average was used. The error bars were calculated by dividing the standard deviation by the square root of the experimental counts.

### 2.8. Swelling Ratio, Water Content, and Water Uptake

The ionogels were incubated in 100 mL water at 25 °C for 24 h. The swollen gels (hydrogels) were removed from the water, and the surface water was gently removed. After gravimetric measurement, the hydrogels were freeze-dried for 12 h at −50 °C under vacuum to obtain dry cryogels. The swelling ratio (SR) of the cellulose gels was measured gravimetrically following Equation (1) [24,42], whereby m_hydro_ was the weight of the hydrogel after 24 h swelling, and m_cryo_ was the weight of the cryogel after freeze-drying.

(1) $$\mathrm{SR}\left(\%\right)=\frac{m_{\mathrm{hydro}}}{m_{\mathrm{cryo}}}\times 100\%$$

The water content of the swollen hydrogels after swelling in 100 mL water at 25 °C for 24 h was calculated following the Equation (2) [42,43].

(2) $$\mathrm{Water}\left(\%\right)=\frac{m_{\mathrm{hydro}}-m_{\mathrm{cryo}}}{m_{\mathrm{hydro}}}\times 100\%$$

The reswelling behavior (water uptake) was determined by immersing the cryogels in 100 mL water at 25 °C. The swollen gels were weighed every hour for up to 8 h (m_t_) and after 24 h. Before measurement, the water on the surface of the gel was gently removed. The water uptake (WU) was calculated following the Equation (3) [24].

(3) $$\mathrm{WU}\left(\%\right)=\frac{m_{t}-m_{\mathrm{cryo}}}{m_{\mathrm{hydro}}}\times 100\%$$

All experiments were performed in duplicates, and the average values were used. The error bars were calculated by dividing the standard deviation by the square root of the experimental counts.

### 2.9. Solvent Recovery

The ionogels were imbibed in water to obtain hydrogels. The water used for the swelling of the gels was collected to recover [C_4_mpyr][OH]. It was filtered through a filtration paper to ensure that no gel particles remained in the solution, followed by evaporation at 50 °C to reduce the water content. The purity of the obtained [C_4_mpyr][OH] aqueous solution was checked by ^1^H NMR spectroscopy and compared to the spectrum of freshly synthesized [C_4_mpyr][OH].

### 2.10. Compression Test of the Cellulose Gels

Stress–strain curves of the cellulose ionogels and hydrogels were recorded by using an Imada Force Measurement MX2 from IMADA CO., Ltd. (Toyohashi, Aichi, Japan). Before the test, the radius and height of the gels were measured. The compression test was conducted at 20 °C with a crosshead speed of 10 mm min^−1^. The strain of the gels was determined from a distance traveled by the crosshead under the application of stress. The maximum of the stress–strain curves was defined as the maximum compressive strength. The measurement was performed in triplicates, and the average was used. The error bars were calculated by dividing the standard deviation by the square root of the experimental counts.

### 2.11. Antimicrobial Test of Cellulose Gels

Three different cellulose ionogels were prepared, whereby the cellulose and crosslinker (ECH) concentration was varied. Ionogel 1 was prepared by using 3.5 wt% cellulose and 10 eq. ECH. For ionogel 2, 3.5 wt% cellulose and 3 eq. ECH was used, and ionogel 3 was prepared by employing 5 wt% cellulose and 3 eq. ECH. The antimicrobial activity of ionogel 1–3 (containing [C_4_mpyr][OH]) and one hydrogel prepared by 5 wt% cellulose with 3 eq. ECH after swelling in 180 mL water for 24 h (not containing [C_4_mpyr][OH]) was investigated by the disk diffusion method. *Escherichia coli* (*E. coli B/r*) and *B. subtilis* were used as bacteria. Sterile distilled water on a paper disk was used as a negative control, and ampicillin 10 μg was used as a positive control. The bacterial suspension in saline was prepared equivalent to 1–2 × 10^8^ CFU mL^−1^. A sterile cotton swab was dipped in the bacterial suspension and spread on the Mueller–Hinton agar. The negative and positive control disks were placed as well as 0.1 g of the cellulose gels. It was incubated at 37 °C for 16 h. The experiment was performed in duplicate, and the diameter of the inhibition zone was measured. The error bars were calculated by dividing the standard deviation by the square root of the experimental counts.

## 3. Results and Discussion

### 3.1. Gelation Test

**Figure 1:**
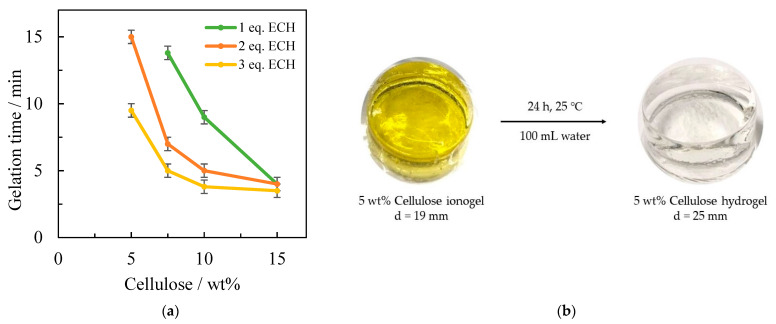
(**a**) Gelation time of cellulose dissolved in aqueous [C_4_mpyr][OH] (50 wt% water) solution as a function of cellulose concentration for different crosslinker (ECH) concentrations at 25 °C. (**b**) Example of a cellulose ionogel (left) and hydrogels (right) made by using 5% cellulose with their diameter (d).

Cellulose can be cross-linked by the introduction of a crosslinker. In this study, ECH was employed as a crosslinker. The proposed mechanism of cross-linking is shown in Scheme S1. In an etherification reaction, the hydroxyl groups of cellulose were chemically cross-linked. In Figure 1, the gelation time depending on the cellulose concentration is shown. The gelation time was highly dependent on the cellulose concentration and crosslinker concentration. After the introduction of the crosslinker, it was noticed after only a few minutes that the viscosity of all solutions increased, indicating that gelation was in progress. The gelation time, mentioned in Figure 1, is the time required for the preparation of self-standing gels and defines the point of complete gelation.

The average time of three experiments was used, whereby the time differences between each experiment were within ±30 s. Higher crosslinker concentration led to faster gelation because of the density increase of chemically cross-linked units in the cellulose network. A nearby exponential relationship between cellulose concentration and gelation time was observed (Figure 1). Low cellulose concentration led to longer gelation time, whereby complete gelation of 5 wt% cellulose with 1 eq. ECH was detected after 1.5 h (not shown in Figure 1). At high cellulose concentration (15 wt%), it was observed that an increase in crosslinker concentration did not result in significantly faster gelation. It is conceivable that in addition to chemical cross-linking, physical cross-linking and chain entangling of cellulose molecules also takes place, which is more pronounced at higher cellulose concentrations (around 15 wt%) so that an increased ECH concentration has only a slight effect on the gelation time. Below 15 wt% cellulose, physical cross-linking is weaker, so that a change in ECH concentration has a significant effect on gelation.

Since 3 eq. resulted in the fastest gelation at 25 °C, which was within 10 min for 5–15 wt% cellulose, an ECH-AGU mole ratio of 3 was used in the following experiments. Although in other studies, a temperature of 50 °C [24] or 5 °C [25] for 24 h was applied for the gelation and curing, this study shows that complete gelation is possible at ambient conditions (25 °C) within 10 min.

As a control, [C_4_mpyr][OH] (50 wt% water) was mixed with ECH, and no gelation was observed. Even after adding 30 wt% ECH and standing for 24 h, no gelation was observed, which leads to the conclusion that no cross-linking or polymerization reaction occurs between [C_4_mpyr][OH] and ECH.

### 3.2. Appearance of the Cellulose Gels

The texture and the size of the gels were highly dependent on the employed cellulose concentration. An example of the gel texture and color before imbibing water (ionogel) and after imbibing water (hydrogel) is given in Figure 1b. Cellulose hydrogels prepared with lower cellulose concentration tend to form more elastic, flexible hydrogels with a larger size. Meanwhile, hydrogels prepared with a higher cellulose concentration were more brittle and had a smaller size. All the gels showed very high transparency regardless of the cellulose concentration. When a piece of writing was placed under the gel (ionogel or hydrogel), the letters could be seen clearly.

The ionogels were yellow in color, and hydrogels were colorless regardless of the cellulose concentration. It is conceivable that the ionogel color comes from the employed cellulose since the color of the solution has already changed to yellow during the dissolution process. An example of cellulose dissolution is shown in Figure S1. In this study, the colorless Avicel^®^cellulose was employed, which has a lignin content of 0.4% [44]. The small amount of dissolved lignin may have led to the color change. After allowing the ionogels to swell in water, colorless hydrogels were obtained. When imbibing water, [C_4_mpyr][OH] was extracted from the gel; at the same time, possibly some amount of lignin was also separated from the gel. This means that the very small amount of lignin was soluble in [C_4_mpyr][OH] aqueous solution and did not cross-link with cellulose or the lignin molecules with each other.

### 3.3. [C_4_mpyr][OH]-Content of the Cellulose Gels

**Figure 2:**
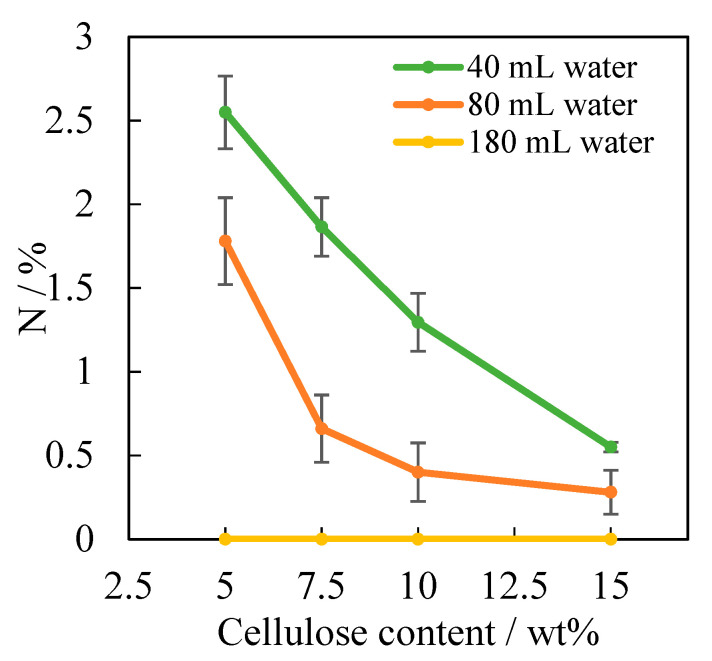
Nitrogen content of cellulose hydrogels swollen in 40, 80, or 180 mL of water for 24 h at 25 °C, and then dried to cryogels.

The [C_4_mpyr][OH]-content of the cellulose gels was determined by measuring the nitrogen content determined by elemental analysis from the respective cryogels, shown in Figure 2. Since neither cellulose nor ECH contains nitrogen, the values can be interpreted as [C_4_mpyr][OH] concentration. Figure 2 shows that [C_4_mpyr][OH] can be extracted from the gel by allowing the gel to swell in water. The degree of extraction can be controlled by adjusting the amount of water in the swelling process. When using 180 mL water, [C_4_mpyr][OH] was completely extracted from the gel. In contrast, the usage of 40 or 80 mL water leads to the remaining of [C_4_mpyr][OH] in the gel. A low cellulose concentration, e.g., 5 wt% cellulose, leads to a higher [C_4_mpyr][OH] content in the gel, while a high cellulose concentration, e.g., 15 wt% cellulose, leads to a higher [C_4_mpyr][OH] extraction. Higher cellulose concentrations lead to more pronounced physical cross-linking, which can result in the [C_4_mpyr][OH] ions being more easily extracted from the gel.

### 3.4. Swelling Ratio, Water Content, and Water Uptake

**Figure 3:**
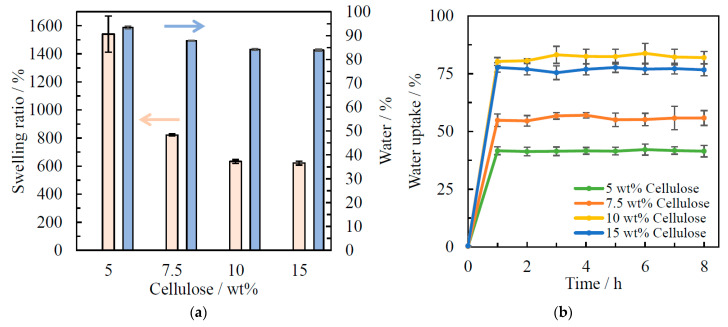
(**a**) Swelling ratio of the cellulose gels after swelling in 100 mL water for 24 h at 25 °C as a function of the cellulose concentration (left, orange). The water content of the hydrogels that were swollen under the same conditions is plotted on the secondary vertical axes (right, blue). (**b**) Water uptake of the cellulose gels with varying cellulose content after swelling in 100 mL water for up to 8 h at 25 °C.

The swelling and re-swelling behavior of the cellulose gels in water at 25 °C was investigated as a function of the cellulose concentration. In Figure 3a, on the left vertical axis, the swelling ratio is plotted against the cellulose concentration. For all the gels, 24 h swelling in water represents the state of equilibrium. The cellulose concentration strongly influences the swelling behavior. A higher cellulose concentration resulted in less swelling, confirming the results from the literature [45]. With increasing cellulose concentration, the physical cross-linking of the polymer chain increases by hydrogen bonding and chain entanglements which lead to a more stable system and a decrease in swelling ratio. However, it is noticeable that the gels with a cellulose concentration of 15 wt% did not follow this trend. The difference between the swelling ratio of 10 and 15 wt% cellulose gels was only 7%. It is conceivable that an equilibrium of physical cross-linking already occurred at 10 wt%. Therefore, increasing the cellulose concentration had no major effect on the swelling behavior. Overall, all the gels showed high swelling in water.

The water content of the swollen hydrogels is shown in Figure 3a on the right vertical axis. A similar tendency to that of the swelling ratio was found for the water content. An increase in the cellulose concentration led to a decrease in the water content. Water enters the gel during the swelling process, and [C_4_mpyr][OH] is flushed out of the gel. Thus, higher swelling leads to more water in the gel. Compared to the literature [43] where the water content of ECH-crosslinked cellulose hydrogels (4 wt% cellulose) was measured, the water content of the gel with 5 wt% cellulose shown in Figure 3 is in a similar order of magnitude and only about 5 lower, which can be explained by the difference in cellulose concentration. Interestingly, in the literature [43], cross-linking took place at 0 °C for 2 h, followed by curing at 60 °C for 2 h. Although different temperature and time conditions were used in other studies, similar results were obtained in this study. It can be concluded that with a much shorter cross-linking procedure (3–10 min) at 25 °C without specific curing, gels with similar properties can be prepared.

The kinetics of water uptake of the hydrogels dried under vacuum (cryogels) was measured at 25 °C and is shown in Figure 3b. The water uptake represented a re-swelling process. For all gels, regardless of the cellulose concentration, the re-swelling reached equilibrium after only 1 h and did not change after a total of 24 h of swelling (not shown in the graph). Gels with a higher cellulose concentration resulted in increased water absorption. This tendency has already been observed in the literature [45]. The reason for this is the drying of the hydrogels.

During the drying process, in this case, freeze-drying, the water was removed from the hydrogel by sublimation, resulting in dense cryogels. Here, the average distance between the cross-linking sites was reduced, which led to a considerable increase in the original cross-linking density and thus to a significant reduction in water absorption. A higher cellulose concentration (more cross-linking) resulted in increased stability of the gels and less dense gels. Gels with a low cellulose concentration, which were less stable, became intensely dense due to the drying process. It is conceivable that gels with a higher cellulose concentration have more intact pores due to the stability to which water can adhere better. However, it must be mentioned here that the drying process of the hydrogels has a major influence on re-swelling. In other studies, it was shown that drying hydrogels at elevated temperatures (50 °C for 20 h) [45] resulted in much higher water absorption, which also followed the trend of cellulose concentration. A drying process near room temperature should be considered in future studies.

In summary, the swelling and re-swelling behaviors are inverse, and by adjusting the cellulose concentration, these properties can be controlled. A low cellulose concentration should be chosen when high initial swelling is desired. If the aim is to achieve the greatest possible re-swelling, a higher cellulose concentration should be selected. By implementing swelling and drying cycles, the gels can be used several times to absorb liquid, which is interesting from the point of view of sustainability. Use of these gels, e.g., in the hygiene field as reusable diapers or reusable wound dressings, are conceivable.

### 3.5. Recovery of [C_4_mpyr][OH] Aqueous Solution

The solvent was collected during the swelling of the gels. Comparing the ^1^H NMR spectra of the collected samples with freshly synthesized [C_4_mpyr][OH], no signal change was observable (Figure S2). This confirms that extraction of [C_4_mpyr][OH] takes place in the swelling process, and since no new signals were found, it can be assumed that the crosslinker ECH reacts completely with the hydroxyl groups of cellulose without water-soluble by-products. Furthermore, by swelling in water, an environmentally benign recycling process was possible, where [C_4_mpyr][OH] can be collected and recycled as cellulose solvent.

### 3.6. Compression Test of the Cellulose Gels

**Figure 4:**
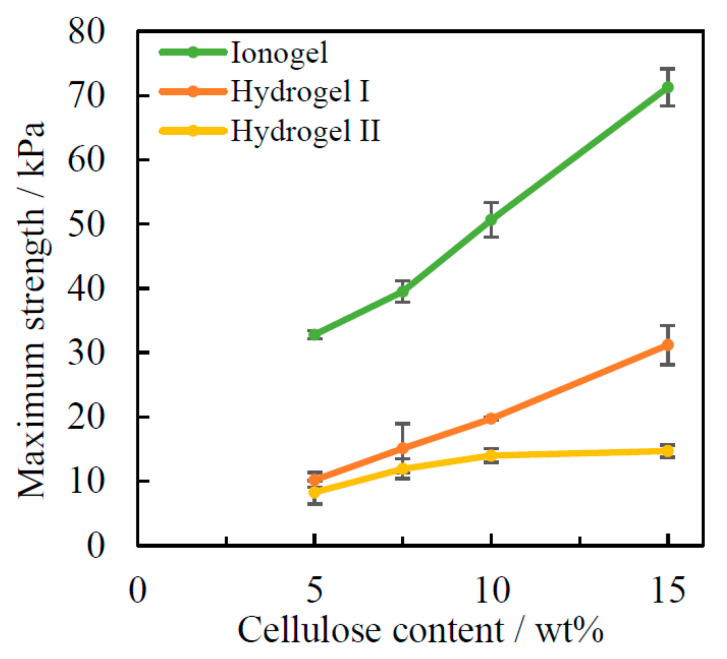
Maximum compressive strength of cellulose ionogels and hydrogels. The hydrogels were swollen in water for 24 h in 40 mL water (Hydrogel I) or 80 mL water (Hydrogel II) at 25 °C.

The maximum compressive strength of the ionogels and hydrogels with varying cellulose concentrations is shown in Figure 4. The maximum compressive strength, also called ultimate strength, is the maximum stress that a material can withstand while remaining in the elastic (reversible) deformation range. First, the stress–strain curves of each gel were measured (Figures S3–S5). At the point where the gels started to break, a sudden decrease in stress was observed, followed by an increase in stress until the next breaking. The point right before the first breaking was chosen as the limit of the stress–strain curves and as the maximum strength (Figure 4). The compressive stress of the cellulose gels increased with increasing cellulose concentration (Figure 4). This result confirms the findings known from the literature that higher biopolymer concentration leads to an increase in compressive stress [46]. Furthermore, the ionogels showed higher compressive resistance than the hydrogels. It is conceivable that the cation and anion of [C_4_mpyr][OH] are building hydrogen bonds when dissolving cellulose which led to an increase in stability. The hydrogels also contained a large amount of water (Figure 3) which led to more flexibility and elasticity but also to lower overall stability, i.e., compressive strength.

The hydrogels were swollen in water for 24 h in 40 mL water (Hydrogel I) or 80 mL water (Hydrogel II) before the compressive measurement. In this process, water was imbibed into the gel, and [C_4_mpyr][OH] was extracted to a certain extent from the gel. The [C_4_mpyr][OH]-concentration is assumed to be relative to the nitrogen concentration measured by elemental analysis (Figure 2), showing that Hydrogel I contained between 25 and 50% more [C_4_mpyr][OH] than Hydrogel II depending on the cellulose concentration. Thus, a higher proportion of [C_4_mpyr][OH] resulted in higher strength.

The comparison of this mechanical study to other previously published studies is complex, as the source of cellulose has a strong influence on the mechanical properties through a different degree of polymerization (DP). For example, bacterial cellulose [47] can have a DP of about 15,000 depending on the incubation conditions, whereas Avicel^®^cellulose [48] has a DP of 240. A higher DP leads to a more stable system and higher maximum strength [49]. Furthermore, the choice and concentration of cross-linking have a great influence on the mechanical stability of the gels. Higher crosslinker concentration is leading to higher strength [49]. The gel preparation also affects the stability, e.g., multiply cross-linking also leads to higher stability [25].

Overall, the gels produced in this study were soft, especially the hydrogels, and despite the relatively low maximum compressive strength compared to studies that used higher DP, higher crosslinker concentration, or double cross-linking, it was still possible to handle the gels without breaking. Furthermore, the mechanical properties of the gels could be controlled by initial cellulose concentration and the amount of water used for the swelling. While the prepared gels do not appear to be suitable for high-intensity mechanical stress, e.g., articular cartilage, light to “normal” mechanical stress, such as wound-dressings or general liquid absorption (hygiene products) are conceivable as applications.

### 3.7. Antimicrobial Test of Cellulose Gels

**Figure 5:**
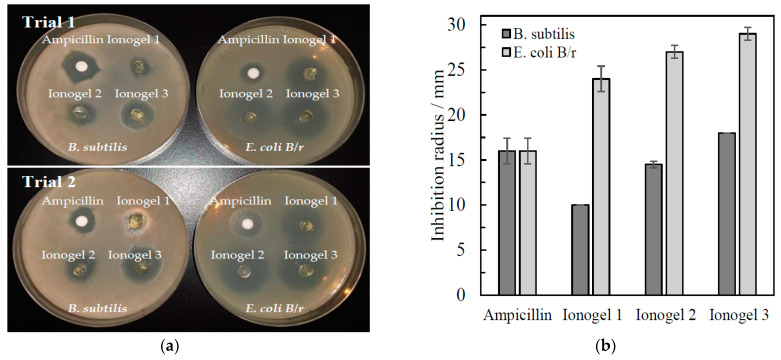
(**a**) Extract from the disk-diffusion test. The antibacterial of ionogels with different cellulose and crosslinker concentration was investigated against *B. subtilis* and *E. coli B/r* after incubation at 37 °C for 16 h. (**b**) Inhibition diameter of ampicillin (positive control) and the ionogels in the disk-diffusion test against the bacteria *B. subtilis* and *E. coli B/r* after incubation at 37 °C for 16 h. Because the tested hydrogels did not show any inhibition, they are not shown in the figure. The cellulose and crosslinker concentration of the ionogels were varied as follows: Ionogel 1 = 3.5 wt% cellulose, 10 eq. ECH; Ionogel 2 = 3.5 wt% cellulose, 3 eq. ECH; Ionogel 3 = 5.0 wt% cellulose, 3 eq. ECH.

The antibacterial activities of cellulose ionogels and hydrogels against *B. subtilis* and *E. coli B/r*, as model representatives for Gram-positive and Gram-negative bacteria, were measured by the disk diffusion method after incubation at 37 °C for 16 h. Both bacteria can be found in wound infections [50]. The results of the disk-diffusion test are shown in Figure 5, Figure S6, and Table S1. All the ionogels showed antimicrobial activity (Figure 5), and the hydrogel did not show any inhibition (Figure S6). The average inhibition zone of all samples in comparison to the positive control (ampicillin) is shown in Figure 5b and Table S1. Interestingly, the antimicrobial strength (inhibition zone) was stronger (wider) by decreasing the amount of crosslinker (10 eq. to 3 eq.) ECH and increasing the cellulose concentration (3.5 wt% to 5 wt%). Ionogel 3 showed the strongest antimicrobial activity, with an average inhibition zone diameter against *B. subtilis* of 18 mm and against *E. coli B/r* 29 mm. As a positive control, the broad-spectrum antibiotic ampicillin was used and showed an inhibition diameter of 16 mm. No inhibition zone was observed for hydrogels and paper as control. This clearly indicates that the antimicrobial activity of ionogels can be attributed to the presence of [C_4_mpyr][OH]. It is conceivable that the cellulose and crosslinker concentrations have an influence on the extraction behavior of [C_4_mpyr][OH] from the ionogel since they change the density of the gel. A lower degree of cross-linking may favor the diffusion of [C_4_mpyr][OH]. Another hypothesis is that besides [C_4_mpyr][OH], another component influences the antibacterial activity. Since a lower crosslinker concentration leads to stronger antibacterial activity (Figure 5b, Ionogel 1 and 2), it can be assumed that the crosslinker itself does not have a great influence on the activity probably due to the extremely fast reaction capacity caused by the ring strain. However, since a higher proportion of cellulose leads to an increased antibacterial effect (Figure 5b, Ionogel 2 and 3), another cause could be cellulose. However, since cellulose is biodegradable [4] by bacteria, pure cellulose cannot, in principle, have any antibacterial effect. In this study, Avicel cellulose was used, which in addition to microcrystalline cellulose as the main compound also contains 0.4% lignin [44]. Aqueous ammonium-based ionic liquids can, for example, convert lignin into aromatic compounds, such as benzoic acid, 2-methoxy-4-(1-propenyl)-phenol, eugenol, benzyl phenyl formats, or 2,3-dimethyl-2-propyl-oxazolidine [51]. Among these degradation products, eugenol, and oxazolidine derivatives have antibacterial properties [52,53]. It is conceivable that [C4mpyr][OH] to a certain extent favors the depolymerization of lignin, which degradation products can have a supporting antibacterial effect.

All in all, the ionogels that are containing [C_4_mpyr][OH] have antimicrobial activity against two bacterial strains that are found in wound infections. Studies using nanosilver as antibacterial agents have measured a maximum inhibition diameter of 23.6 mm for cellulose/nanosilver composite sponges against *E. coli* [54]. The antibacterial strength of the Ionogel 3 used in this study has even a stronger antibacterial activity against *E. coli B/r* with an inhibition diameter of 29 mm. The test result indicates that the antibacterial activity of the ionogels in this study was strong against *E. coli B/r* and *B. subtilis,* making it a suitable candidate as a material for wound dressings that is resistant to bacterial infections. In particular, *E. coli* is a multi-resistant bacterium, which means insensitivity to a broad range of antibiotics, causing an increasing demand for new antibiotics [55]. Ionogels that contained [C_4_mpyr][OH] showed stronger antibacterial activity against *E. coli B/r* than *B. subtilis* (inhibition diameter ionogel 3: 29 mm < 18 mm), making the ionogels prepared in this study a new promising approach as an antibacterial material in wound-dressing, and especially against the antibiotic-resistant strain *E. coli*.

## 4. Conclusions

A simple and rapid one-pot method for the preparation of highly transparent cellulose gels under mild conditions was developed by employing unsubstituted cellulose, [C_4_mpyr][OH] in an aqueous solution as the solvent and ECH as the crosslinker. Cellulose gels can be prepared within a total time of 30 min, including dissolution and gelation at room temperature, by selecting a relatively small ECH-AGU mole ratio of 3 eq. The gelation time was controlled by the crosslinker concentration.

The properties of the cellulose gels were investigated, and by adjustment of the cellulose concentration and employed water, the swelling ratio, water uptake, and mechanical strength under compression could be controlled. Furthermore, the cellulose ionogels showed antibacterial activity against *B. subtilis* and *E. coli B/r* due to the presence of [C_4_mpyr][OH]. Thus, these ionogels can eventually contribute as biomaterials resistant to wound infections in the future. Cellulose hydrogels, which consist only of cross-linked cellulose and water, were prepared by simple extraction of [C_4_mpyr][OH] from the gel (swelling in water). The remaining concentration of [C_4_mpyr][OH] in the hydrogel can be controlled by choosing a specific volume of water. The extracted [C_4_mpyr][OH] was successfully recovered so that an environmentally benign recycling process and re-use of the solvent were possible. By choosing the cellulose concentration and the amount of water in the swelling process, the maximum compressive strength of the hydrogels was also controlled. In this study, [C_4_mpyr][OH] in an aqueous solution was not only used as a solvent for cellulose but also as a mild antibacterial agent.

“Designer gels” can be made by adjustment of the cellulose concentration and volume of the water employed for swelling. Very soft gels can be prepared by using a smaller cellulose concentration (5 or 7.5 wt%). Soft to slightly brittle gels that resist more mechanical stress can be prepared by employing a higher amount of cellulose (10 or 15 wt%). In this study, we developed a simple and fast cellulose gel preparation method that allows solvent recovery. The complete elimination of heating and cooling techniques for dissolution of unmodified cellulose, gelation, and curing of the gels in a short time is an innovative approach to prepare cellulose hydrogels in an environmentally benign manner. The gels produced in this study can be considered sustainable or more sustainable than other gels, as their preparation requires significantly less energy (no heating and cooling) and should be biodegradable due to the main component being cellulose. It is conceivable that the prepared gels can contribute as a sustainable, more environmentally friendly alternative material to absorb liquids in various areas, e.g., hygiene products or wound dressings.

## Data Availability

The data presented in this study are available on request from the corresponding author.

## Supplementary Materials

The following are available online at https://www.mdpi.com/article/10.3390/polym13121942/s1. Scheme S1: Proposed cross-linking mechanism of cellulose with epichlorohydrin under alkaline condition. Figure S1: Dissolution example of cellulose in [C_4_mpyr][OH] aqueous solution. Figure S2: 1H NMR of [C_4_mpyr][OH] after synthesis (top) and [C_4_mpyr][OH] recovered from gels (bottom). Figure S3: Stress–strain curves of ionogels with different cellulose concentration. Figure S4: Stress–strain curves of hydrogels with different cellulose concentration swollen in 40 mL water for 24 h at 25 °C. Figure S5: Stress–strain curves of hydrogels with different cellulose concentration swollen in 80 mL water for 24 h at 25 °C. Figure S6: Disk-diffusion test of ionogels and hydrogels against *B. subtilis* and *E. coli* B/r. after incubation at 37 °C for 16 h. As positive control ampicillin and as negative control water was used. Table S1: Inhibition zone of ionogels, hydrogel, and Ampicillin against *B. subtilis* and *E. coli* B/r. after incubation at 37 °C for 16 h.
