# Supplementary material for: Simple and Fast One-Pot Cellulose Gel Preparation in Aqueous Pyrrolidinium Hydroxide Solution–Cellulose Solvent and Antibacterial Agent
Source: Polymers (Basel). 2021 Jun 11;13(12):1942. doi: 10.3390/polym13121942 (PMC8230700; doi:10.3390/polym13121942)
Supplement: Supplementary file 1 [file polymers-13-01942-s001.zip › polymers-1242312-supplementary.pdf]

polymers

Supplementary Information

Simple and Fast One-Pot Cellulose Gel Preparation in Aqueous Pyrrolidinium Hydroxide Solution–Cellulose Solvent and Antibacterial Agent

Elisabeth R. D. Seiler, Kohei Koyama, Tomoyuki Iijima, Tamao Saito, Yuko Takeoka, Masahiro Rikukawa and Masahiro Yoshizawa-Fujita *

Department of Materials and Life Sciences, Sophia University, 7-1 Kioi-cho, Chiyoda-ku, Tokyo 102-8554, Japan; e-seiler@eagle.sophia.ac.jp (E.R.D.S.); kouheihunpty@icloud.com (K.K.); jima-tomo@eagle.sophia.ac.jp (T.I.); tasaito@sophia.ac.jp (T.S.), y-tabuch@sophia.ac.jp (Y.T.); m-rikuka@sophia.ac.jp (M.R.)

* Correspondence: masahi-f@sophia.ac.jp

1. Cross-linking mechanism

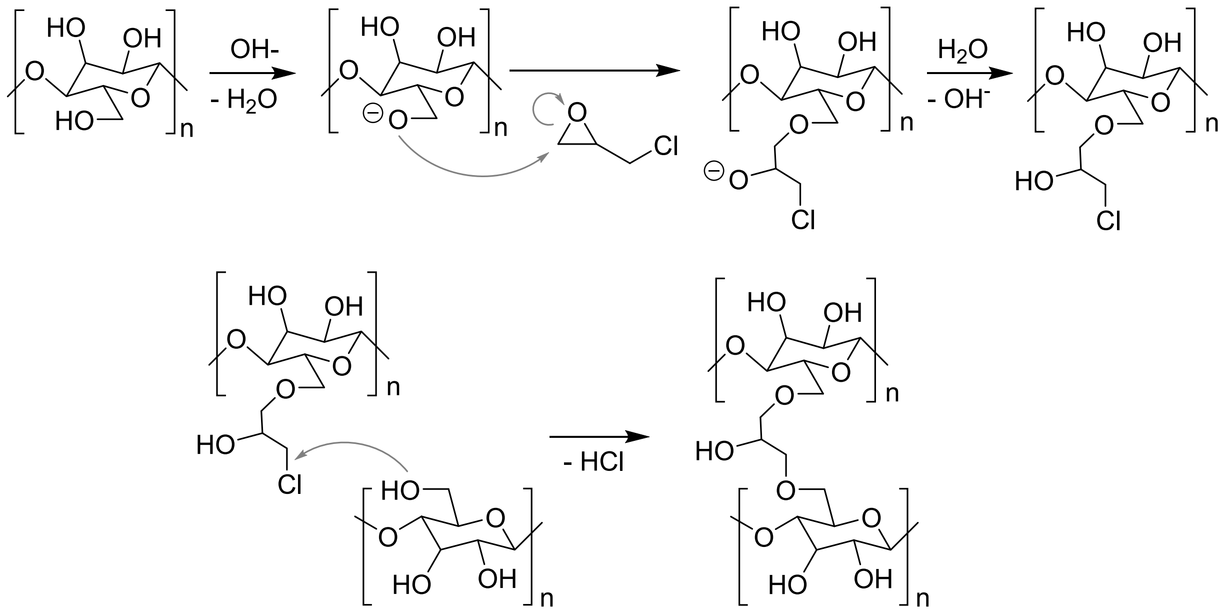

Scheme S1. Proposed crosslinking mechanism of cellulose with epichlorohydrin under alkaline condition.

2. Dissolution of cellulose

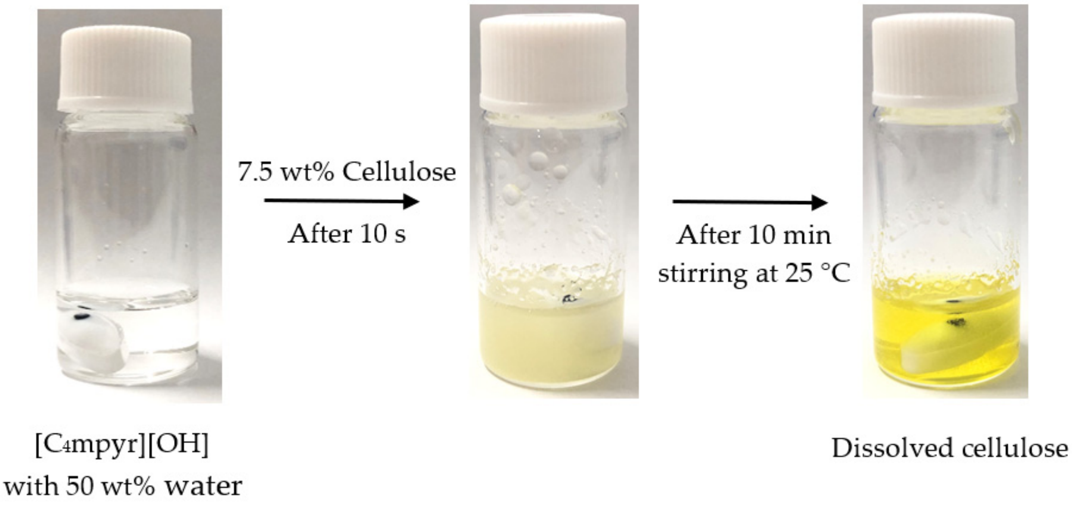

Figure S1. Dissolution example of cellulose in [C4mpyr][OH] aqueous solution.

3 1.H-NMR of regenerated [C4mpyr][OH]

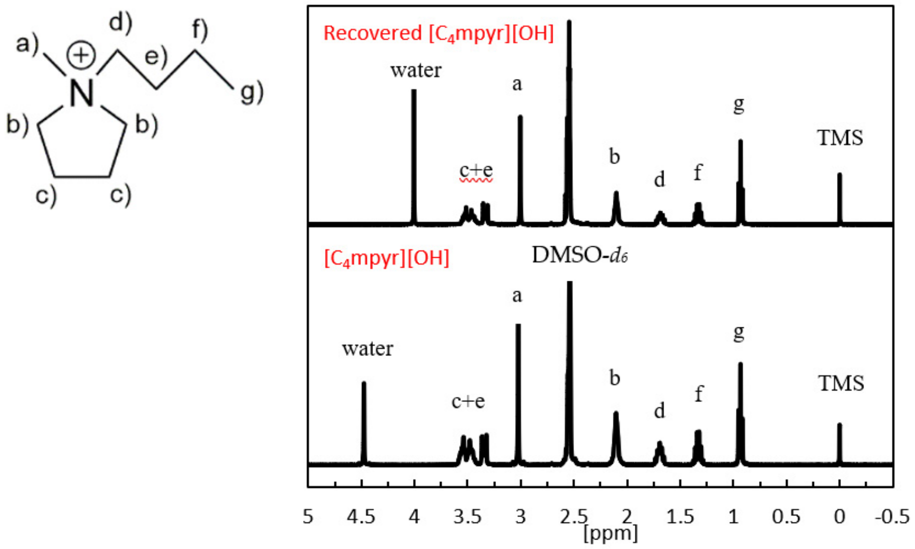

Figure S2. ^{1}H NMR of [C4mpyr][OH] after synthesis (bottom) and [C4mpyr][OH] recovered from gels (top).

4. Compression test

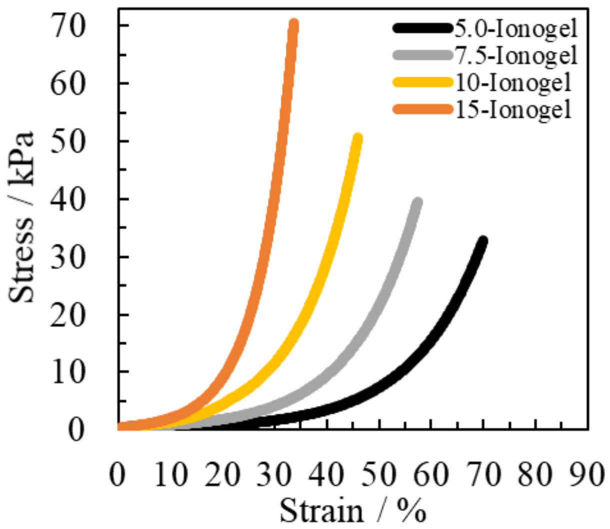

Figure S3. Stress-strain curves of ionogels with different cellulose concentration.

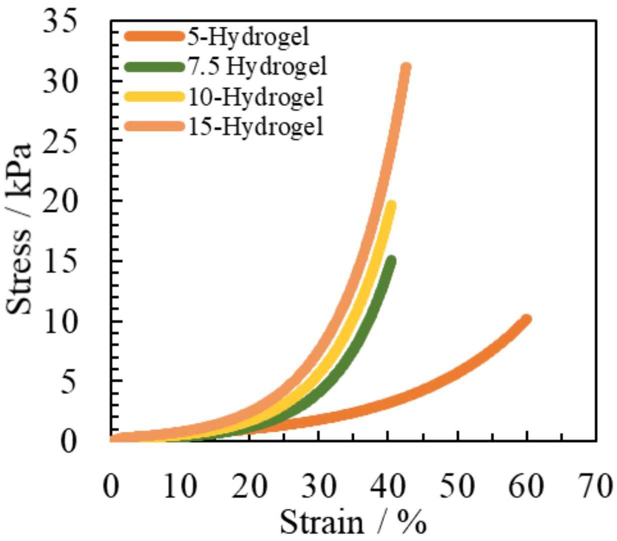

Figure S4. Stress-strain curves of hydrogels with different cellulose concentration swollen in 40 mL water for 24 h at 25 °C.

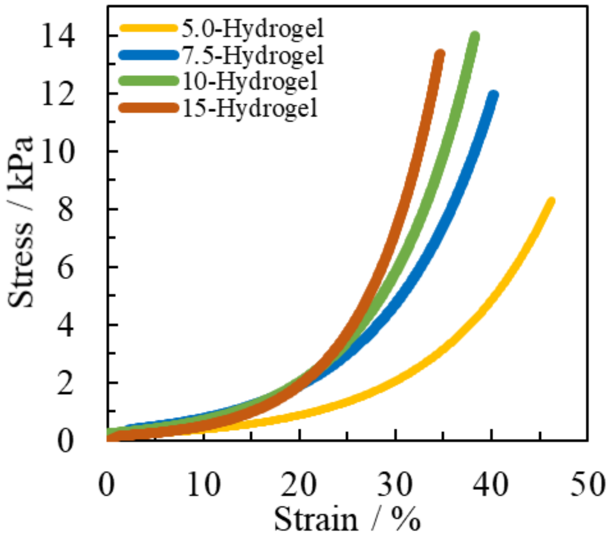

Figure S5. Stress-strain curves of hydrogels with different cellulose concentration swollen in 80 mL water for 24 h at 25 °C.

5. Antibacterial Test (Disc-diffusion Test against B. subtilis and E. coli B/r)

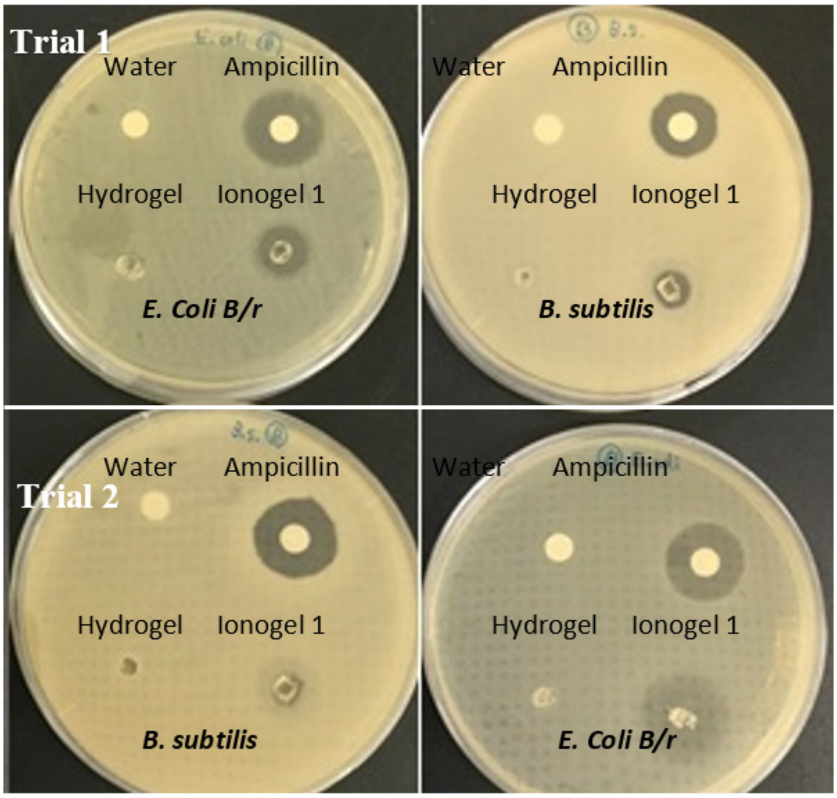

Figure S6. Disk-diffusion test of ionogels and hydrogels against B. subtilis and E. coli b/r. after incubation at 37 °C for 16 h. As positive control ampicillin and as negative control water was used.

Table S1. Inhibition zone of ionogels, hydrogel and Ampicillin against B. subtilis and E. coli B/r. after incubation at 37 °C for 16 h.

				Inhibition Zone / mm		
**Sample Name**	**Bacillus**	**Cellulose / wt%**	**Crosslinker / eq.**	**Trial 1**	**Trial 2**	**Average**
Ampicillin	B. subtilis	0	0	18	14	16
Ionogel 1		5	10	10	10	10
Ionogel 2		3.5	10	15	14	14.5
Ionogel 3		5	3	18	18	18
Hydrogel		5	3	0	0	0
Ampicillin	E. coli B/r	0	0	14	18	16
Ionogel 1		5	10	22	26	24
Ionogel 2		3.5	10	26	28	27
Ionogel 3		5	3	28	30	29
Hydrogel		5	3	0	0	0
